# Establishing Bipotential Human Lung Organoid Culture System and Differentiation to Generate Mature Alveolar and Airway Organoids

**DOI:** 10.21769/BioProtoc.4657

**Published:** 2023-04-20

**Authors:** Man Chun Chiu, Cun Li, Yifei Yu, Xiaojuan Liu, Jingjing Huang, Zhixin Wan, Kwok Yung Yuen, Jie Zhou

**Affiliations:** 1 Department of Microbiology, School of Clinical Medicine, Li Ka Shing Faculty of Medicine, The University of Hong Kong, Hong Kong, China; 2 State Key Laboratory of Emerging Infectious Diseases, The University of Hong Kong, Hong Kong, China; 3 Centre for Virology, Vaccinology and Therapeutics, Hong Kong Science and Technology Park, Hong Kong, China

**Keywords:** Lung organoids, Airway organoids, Alveolar organoids, Long-term expansion, Proximal differentiation, Distal differentiation

## Abstract

A robust in vitro model of the human respiratory epithelium, including the alveolar and the airway epithelium, is essential for understanding the biology and pathology of the human respiratory system. We previously described a protocol to derive human lung organoids from primary lung tissues. We now describe a protocol to induce bidirectional differentiation to generate mature alveolar or airway organoids. The lung organoids are consecutively expanded for over one year with high stability, while the differentiated alveolar and airway organoids morphologically and functionally simulate the human alveolar and airway epithelium to a near-physiological level. Thus, we establish a robust organoid culture system of the entire human respiratory epithelium, the first two-phase bipotential organoid culture system that enables long-term expansion and bidirectional differentiation of respiratory epithelial cells. The long-term expandable lung organoids and differentiated organoids generate a stable and renewable source of respiratory epithelial cells, enabling scientists to reconstruct and expand the human respiratory epithelium in culture dishes. The respiratory organoid system provides a unique and physiologically active in vitro model of the human respiratory epithelium for various applications, including studying respiratory viral infection, disease modeling, drug screening, and pre-clinical testing.

Graphical abstract

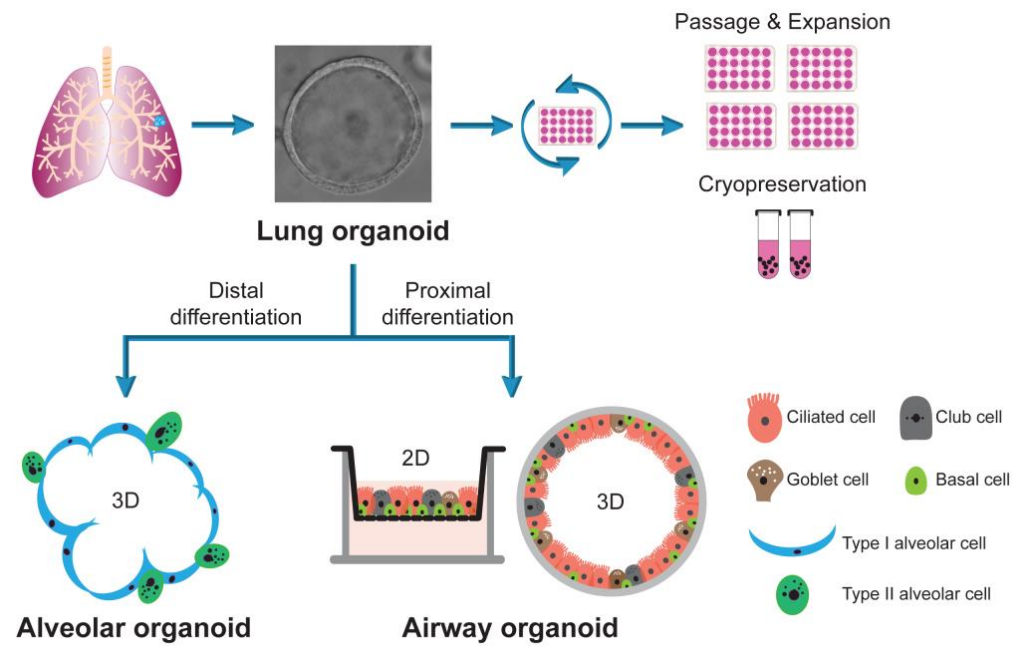

## Background

Recent advances in stem cell technology enable the generation of *mini-organs* or *organs-in-a-dish*, known as organoids. By definition, organoids are three-dimensional cultures derived from stem cells; they mimic the in vivo architecture and functionality of the corresponding tissue or organ (Lancaster and Knoblich, 2014; M. Li and Izpisua Belmonte, 2019; Schutgens and Clevers, 2020). Organoids have become a robust and innovative tool for modeling developmental biology, physiology, and pathology.

Organoids can be derived from either pluripotent stem cells or adult stem cells (ASC). When provided with appropriate niche factors, ASCs isolated from tissues can self-renew and self-organize into organ-like multicellular clusters composed of multiple tissue-specific cell types, which morphologically and functionally simulate the in vivo counterparts. The generation of the first ASC-derived organoid, the human intestinal organoid, was reported in 2009 (Sato et al., 2009 and 2011). Afterward, ASC-derived organoids were established for a variety of human organs and tissues, including the prostate (Chua et al., 2014; Karthaus et al., 2014), liver (Huch et al., 2013b; Hu et al., 2018), stomach (Wroblewski et al., 2015; Schlaermann et al., 2016), pancreas (Huch et al., 2013a), mammary gland (Sachs et al., 2018), and lung (Zhou et al., 2018; Sachs et al., 2019). These ASC-derived organoids retain the fundamental cellular, structural, and functional properties of the native organ and maintain genotypic and phenotypic stability during long-term culture.

The human respiratory tract is lined with two distinct types of epithelia: the airway and the alveolar epithelium. We established the long-term expandable ASC-derived human lung organoid from lung tissues in collaboration with Clevers’ lab (Zhou et al., 2018; Sachs et al., 2019). We further developed a proximal differentiation protocol and generated 3D and 2D airway organoids that morphologically and functionally phenocopy the airway epithelium to a near-physiological level (Zhou et al., 2018). A detailed protocol describing the derivation of lung organoids and the generation of differentiated airway organoids was published recently (C. Li et al., 2022). However, derivation of the alveolar epithelium, which consists of type 1 and type 2 alveolar epithelial cells (AT1 and AT2, respectively), from the lung organoids remained elusive.

We recently reported a bipotential lung organoid culture system that can generate both airway and alveolar epithelial cells (Chiu et al., 2022). Here, we provide a detailed protocol to generate 3D alveolar organoids from long-term expanding human lung organoids, the same source for generating airway organoids as previously reported. We also describe a protocol to generate optimized 2D airway organoids from lung organoids, which mimic the airway epithelium more favorably. Altogether, we established a bipotential lung organoid culture system that could enable bidirectional differentiation into alveolar organoids upon distal differentiation or airway organoids upon proximal differentiation. The lung organoids serve as a stable source for long-term expansion, while differentiated airway and alveolar organoids faithfully phenocopy the human airway and alveolar epithelium, respectively. These organoids are robust and physiologically active tools that are applicable to various experimental manipulations, to explore the biology and pathology of the human lungs.

## Materials and Reagents

1. Nunc 15 and 50 mL conical sterile polypropylene centrifuge tubes (Thermo Scientific, catalog numbers: 339650, 339652)
2. Surgical scalpel blade No. 22 (Swann-Morton, catalog number: 0508)
3. 100 mm TC-treated culture dish (Corning, catalog number: 430167)
4. T175 cell culture flask (Greiner Bio-One, catalog number: 661175)
5. Nunc non-treated 24-well plate (Thermo Scientific, catalog number: 144530)
6. Nunclon Sphera 24-well plate (Thermo Scientific, catalog number: 174930)
7. Costar 6.5 and 12 mm Transwell^®^, 0.4 μm pore polyester membrane inserts (Stem Cell Technologies, catalog numbers: 38024, 38023)
8. 40 and 100 μm cell strainer (Falcon, catalog numbers: 352340, 352360)
9. Steritop threaded bottle top filter (0.22 μm) (Merck Millipore, catalog number: SCGPS01RE)
10. 10/100/1,000 μL QSP low retention filtered pipette tips (Thermo Scientific, catalog numbers: TFLR102-10-Q, TFLR113-100-Q, TFLR1121000-Q)
11. 5/10/15 mL Stripette serological pipettes (Corning, catalog numbers: 4487, 4488, 4489)
12. Pasteur pipettes length *ca*. 225 mm (Brand, catalog number: 747720)
13. 5 mL round bottom polystyrene test tube (Falcon, catalog number: 352052)
14. β-BODIPY^TM^ FL C12-HPC (Invitrogen, catalog number: D3792)
15. LysoTracker^TM^ red DND-99 (Invitrogen, catalog number: L7528)
16. Matrigel growth factor–reduced (GFR) basement membrane matrix, phenol red–free, LDEV-free (Corning, catalog number: 356231)
17. Advanced DMEM/F-12 (Gibco, catalog number: 12634010)
18. DMEM (Gibco, catalog number: 10569010)
19. Zeocin^TM^ selection reagent (100 mg/mL) (Gibco, catalog number: R25005)
20. Geneticin^TM^ selective antibiotic (G418 sulfate) (50 mg/mL) (Gibco, catalog number: 10131035)
21. HEPES (Gibco, catalog number: 15630056)
22. GlutaMAX supplement (Gibco, catalog number: 35050061)
23. Penicillin-Streptomycin (Gibco, catalog number: 15140122)
24. Recombinant human Rspondin1 (Stem Cell Technologies, catalog number: 78213; Peprotech, catalog number: 120-38)
25. Rspondin1 expressing 293T cell line (Sigma-Aldrich, catalog number: SCC111)
26. Recombinant human Noggin (Stem Cell Technologies, catalog number: 78060; Peprotech, catalog number: 120-10C)
27. B-27 supplement (50×), serum-free (Gibco, catalog number: 17504044)
28. N-Acetyl-L-cysteine (Sigma-Aldrich, catalog number: A9165)
29. Nicotinamide (Sigma-Aldrich, catalog number: N0636)
30. Y-27632 dihydrochloride (Tocris, catalog number: 1254)
31. A 83-01 (Tocris, catalog number: 2939)
32. SB 202190 (Sigma-Aldrich, catalog number: S7067)
33. Recombinant human KGF (FGF-7) (Peprotech, catalog number: 100-19)
34. Recombinant human FGF-10 (Peprotech, catalog number: 100-26)
35. Recombinant human heregulin β-1 (Peprotech, catalog number: 100-03)
36. Primocin (InvivoGen, catalog number: ant-pm-1)
37. Dexamethasone (Tocris, catalog number: 1126)
38. 8-Bromo-cAMP, sodium salt (Tocris, catalog number: 1140)
39. IBMX (Tocris, catalog number: 2845)
40. CHIR 99021 (Tocris, catalog number: 4423)
41. Recombinant murine WNT3A (Peprotech, catalog number: 315-20)
42. L WNT3A cell line (ATCC, catalog number: CRL-2647)
43. PneumaCult-ALI medium (StemCell Technologies, catalog number: 05001)
44. Heparin solution (StemCell Technologies, catalog number: 7980)
45. Hydrocortisone stock solution (StemCell Technologies, catalog number: 7925)
46. DAPT (Tocris, catalog number: 2634)
47. PIPES (Sigma-Aldrich, catalog number: P1851)
48. Collagenase from *Clostridium histolyticum* (Sigma-Aldrich, catalog number: C9407)
49. TrypLE select enzyme (10×), no phenol red (Gibco, catalog number: A1217701)
50. Phosphate buffered saline (PBS) (Gibco, catalog number: 10010023)
51. Fetal bovine serum (FBS), qualified, heat inactivated (Gibco, catalog number: 10082147)
52. Buffer EL erythrocyte lysis buffer (Qiagen, catalog number: 79217)
53. UltraPure^TM^ 0.5 M EDTA, pH 8.0 (Invitrogen, catalog number: 15575020)
54. Formaldehyde solution [i.e., 37% paraformaldehyde (PFA)] (Sigma-Aldrich, catalog number: 252549)
55. Triton X-100 (Sigma-Aldrich, catalog number: X100)
56. Bovine serum albumin (BSA) (Sigma-Aldrich, catalog number: A9418)
57. DAPT (Sigma-Aldrich, catalog number: D9542)
58. Phalloidin - Atto 647N (Sigma-Aldrich, catalog number: 65906)
59. ProLong^TM^ glass Antifade mountant (Invitrogen, catalog number: P36980)
60. Glutaraldehyde (purchased from electron microscope unit, HKU)
61. Hydrochloric acid (HCl) (Sigma-Aldrich, catalog number: 320331)
62. Sodium hydroxide (NaOH) (Sigma-Aldrich, catalog number: 221465)
63. Basal medium (see Recipes)
64. Expansion medium (see Recipes)
65. Distal differentiation medium (DD medium) (see Recipes)
66. Proximal differentiation medium (PD medium) (see Recipes)
67. Rspondin1 conditioned medium (see Recipes)
68. Noggin conditioned medium (see Recipes)
69. WNT3A conditioned medium (see Recipes)

## Equipment

1. SterilGARD e3 Class II Type A2 biosafety cabinet (Baker Co, catalog number: SG404-INT)
2. Forma Steri-Cycle i160 CO_2_ 165 L incubator (Thermo Fisher Scientific, catalog number: 51030301)
3. New Brunswick Innova^®^ 44/44R stackable incubator shaker (Eppendorf, catalog number: M1282-0002)
4. Centrifuge (Eppendorf, model: 5810R)
5. FE20-Kit FiveEasy benchtop pH meter (Mettler Toledo, model: FE20-KIT)
6. LightCycler 96 Instrument (Roche, catalog number: 381711)
7. Inverted routine microscope (Nikon, model: Eclipse TS100)
8. Confocal microscope (Zeiss, model: LSM 800)
9. Transmission electron microscope (FEI, model: Tecnai G2 20 S-TWIN)
10. Cell analyzer (BD Biosciences, model: FACSCanto II & LSRFortessa)
11. Midi Plus pipetting controllers (Sartorius, model: 710931)
12. Research Plus mechanical pipette (Eppendorf, catalog number: 3123000900)

## Procedure

- **Derivation and expansion of lung organoids**
  1. Preparation
    - Prepare basal medium (Recipe 1) and keep it on ice or at 4 °C.
    - Prepare expansion medium (Recipe 2) and prewarm at 37 °C.
    - Thaw matrigel at 4 °C and keep it on ice.
*Note: Matrigel will solidify at room temperature.*
    - Prewarm a 24-well suspension culture plate in a standard cell culture incubator with 5% CO_2_ and humidified atmosphere at 37 °C.
    - Prepare glass Pasteur pipettes with a narrow opening (≤1 mm) by burning the tips with a flame, such as a Bunsen burner, to narrow the opening from ~1.5 to ≤1 mm. Cool down the Pasteur pipettes. Autoclave the Pasteur pipettes prior to use.
*Note: The burning procedure also aims to smoothen the sharp edge of the glass pipette and reduce cell damage. Any fine tip Pasteur pipette with a narrow opening (≤1 mm) and smooth edge can be used instead.*
    - Rinse the Pasteur pipettes with 2–3 mL of basal medium by pipetting up and down a few times to avoid excessive attachment of cells before using them to mechanically shear the organoids (i.e., steps A3e and B2e).
  2. Derivation of lung organoids
    - Obtain freshly resected lung tissues from patients who underwent surgical operations due to various diseases. Transport the tissue in the cold basal medium (and preferably on ice at 4 °C) and process it as soon as possible.
*Note: The lung tissues/biopsies should be resected from the distal lung region, should be composed of normal tissues adjacent to diseased tissues (with both bronchioles and alveoli), and should be ~0.5 cm in diameter.*
    - Mince the tissue into small pieces (≤1 mm) with a sterile scalpel in a 100 mm cell culture dish. Wash the tissue pieces with 10 mL of cold basal medium and transfer them to a 15 mL centrifuge tube. Centrifuge at 400 *× g* for 5 min at 4 °C. Discard the supernatant.
    - Resuspend the tissue pieces in 8 mL of cold basal medium supplemented with collagenase at a final concentration of 2 mg/mL. Digest the tissue pieces for 30–40 min at 37 °C in a shaking incubator at 120 rpm.
    - Shear the digested tissue pieces by pipetting up and down 20 times using a 10 mL serological pipette. Filter the cell suspension through a 100 μm cell strainer.
    - (Optional) Recover remaining tissue pieces from the cell strainer with cold basal medium and transfer them to a 15 mL centrifuge tube for a second round of mechanical shearing by pipetting and then filtering to increase the cell yield.
    - Add FBS to the flowthrough with a final concentration of 2% to terminate enzymatic digestion. Centrifuge at 400 *× g* for 5 min at 4 °C. Discard the supernatant.
    - (Optional) Resuspend the pellet in 2 mL of erythrocyte lysis buffer and incubate for 5 min at room temperature to remove the red blood cells.
    - Wash the cells with 10 mL of cold basal medium. Centrifuge at 400 *× g* for 5 min at 4 °C. Discard the supernatant.
    - Resuspend the pellet in cold matrigel. Add 80–160 μL of matrigel for cells obtained from a lung tissue of size ~0.5 cm in diameter. Dispense 40 μL of the cell suspension to each well of a prewarmed 24-well suspension culture plate and incubate for 10–15 min at 37 °C in a standard cell culture CO_2_ incubator to let the matrigel droplet solidify.
    - Add 500 μL of expansion medium to each well and incubate the plate in a standard cell culture CO_2_ incubator. Replenish the medium every 2–3 days with caution not to disrupt the matrigel droplets. Observe the organoids and monitor their growth under a light microscope regularly (Figure 1).
*Note: In the initial culture, there are non-epithelial cell types such as lung fibroblasts, which will gradually disappear after 2–3 passages.*
    **Figure 1.**
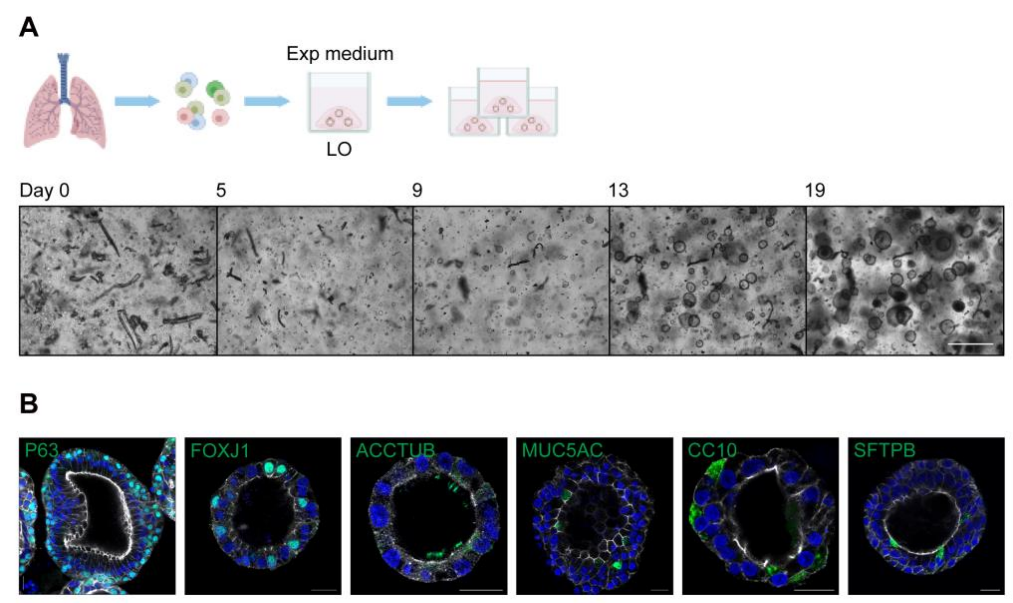
Derivation and expansion of lung organoids. (A). Top, a schematic graph outlines the derivation and expansion of lung organoids (LO) in expansion medium (Exp medium). Bottom, photomicrographs show growing lung organoids derived from isolated lung cells on day 0, 5, 9, 13, and 19. Scale bar = 500 μm. (B). Lung organoids were applied to immunofluorescence staining to label P63+ basal cells, FOXJ1+/ACCTUB+ ciliated cells, MUC5AC+ goblet cells, CC10+ club cells, and SFTPB+ AT2 cells (green). Nuclei and actin filaments were counterstained with DAPI (blue) and Phalloidin-647 (white), respectively. Scale bar = 20 μm.
  3. Expansion of lung organoids
    - Passage and split the lung organoids every 2–3 weeks.
    - Disrupt the matrigel droplets by pipetting up and down the medium 10 times with a 1,000 μL pipette. Transfer the mixture to a 15 mL tube. Top up with cold basal medium to a total volume of 10 mL if several wells of organoids are harvested for passaging. Incubate the mixture on ice for 5 min. Centrifuge at 400 *× g* for 5 min at 4 °C. Discard the supernatant.
    - Wash the organoids once with cold basal medium. Centrifuge at 400 *× g* for 5 min at 4 °C. Discard the supernatant.
    - (Optional) Resuspend the organoids in 1–2 mL of 10× TrypLE and incubate for 5 min at 37 °C to digest the organoids. Add FBS to the organoids with a final concentration of 2% to terminate enzymatic digestion (i.e., add 20 μL of FBS for 1 mL of 10× TrypLE).
*Note: Trypsinization is preferred when the mechanical shearing approach is inadequate to shear the lung organoid into small pieces or when the size of the organoids is highly variable, particularly when subsequent experimentations require organoids of a relatively uniform size.*
    - Shear the organoids into single cells and small cellular clusters by pipetting up and down 20–40 times with a Pasteur pipette.
    - Check the sheared organoids under a light microscope to ensure organoid fragments are small enough (e.g., less than 10 cells). Repeat the mechanical shearing if necessary.
    - Wash the cells with the cold basal medium. Centrifuge at 400 *× g* for 5 min at 4 °C. Discard the supernatant.
    - Resuspend the pellet in an estimated volume of cold matrigel, which enables a split of the organoids with a ratio of 1:2 to 1:10 based on organoid density in the original culture and the methods used for passage. We normally apply a ratio of 1:2 to 1:4 when passaging with mechanical shearing and 1:6 to 1:10 when passaging with enzymatic digestion.
*Note: The cell density should be approximately 2 × 10^4^–4 × 10^4^ cells in each 40 μL of matrigel.*
    - Dispense 40 μL of the cell suspension to each well of a prewarmed 24-well suspension culture plate and incubate for 10–15 min at 37 °C in a standard cell culture CO_2_ incubator to let the matrigel droplet solidify.
    - Add 500 μL of expansion medium to each well and incubate the plate in a standard cell culture CO_2_ incubator. Replenish the medium every 2–3 days with caution not to disrupt the matrigel droplets.
*Note: The lung organoids can be passaged every 2–3 weeks for approximately one year (i.e., 16–24 passages).*
- **Distal differentiation of lung organoids to generate alveolar organoids**
  1. Preparation
    Prepare distal differentiation medium (DD medium) (Recipe 3) and prewarm at 37 °C.
  2. Distal differentiation in suspension culture
    - Culture lung organoids in the expansion medium for 12–14 days to prepare sufficient organoids for differentiation culture (lung organoids from step A3j).
    - Disrupt the matrigel droplets by pipetting up and down the medium 10 times with a 1,000 μL pipette. Transfer the mixture to a 15 mL tube. Top up with cold basal medium to a total volume of 10 mL if several wells of organoids are harvested for passaging. Incubate the mixture on ice for 5–10 min. Centrifuge at 400 *× g* for 5 min at 4 °C. Discard the supernatant.
*Note: Use cold basal medium (keep on ice) to wash the organoids and incubate them on ice to completely remove the matrigel. Residual matrigel may compromise the effect of subsequent suspension culture.*
    - Wash the organoids with cold basal medium. Centrifuge at 400 *× g* for 5 min at 4 °C. Discard the supernatant.
    - Resuspend the organoids in 1–2 mL of 10× TrypLE and incubate for 5 min at 37 °C to digest the organoids. Add FBS with a final concentration of 2% to terminate enzymatic digestion (i.e., add 20 μL of FBS for 1 mL of 10× TrypLE).
    - Shear the organoids into single cells by pipetting up and down 20–40 times using a Pasteur pipette.
    - Check under a light microscope to ensure the organoids are sheared into single cells. Repeat the mechanical shearing if necessary.
    - Wash the cells with 10 mL of cold basal medium. Filter the cell suspension through a 40 μm cell strainer. Centrifuge at 400 *× g* for 5 min at 4 °C. Discard the supernatant.
    - Resuspend the pellet in 1–10 mL of DD medium based on the estimated cell number. Count the number of cells with a hemocytometer under a microscope. Adjust the cell concentration to 2 × 10^5^ per milliliter with DD medium.
    - Dispense 500 μL of cell suspension (1 × 10^5^ cells) to each well of a Nunclon Sphera 24-well suspension culture plate.
*Notes:*
*1) Approximately 2.4 × 10^6^ cells are required for generating a plate of alveolar organoids in 24-well format.*
*2) Do not put too many cells in each well for suspension culture because cells may form aggregates if the density is too high, which may compromise alveolar differentiation.*
*3) Avoid scratching the bottom of the wells during pipetting, which may damage the super low cell attachment surface of the Nunclon Sphera culture plates.*
    - Incubate the cells in a standard cell culture incubator for 10–14 days for maturation (Figure 2). Directly add 100 μL of DD medium to each well every other day without removing the old medium, since evaporation reduces the volume of the medium during incubation. Evenly distribute the cells by gentle shaking before putting the plate back into the incubator.
*Notes:*
*1) Make sure to evenly distribute the cells; they may form aggregates if closely centered in the well, which may compromise alveolar differentiation.*
*2) Organoids with a lumen and thin wall are discernible under a microscope from day 3–4 after suspension cultured in DD medium. After 10 days of distal differentiation culture, the alveolar organoids are mature enough to simulate the native alveolar epithelium, which is applicable for subsequent experimental manipulation.*
*3) The thin and flat AT1 cells are delicate. Pipette gently to avoid disturbing the AT1 morphology during the differentiation culture and downstream procedures.*
    **Figure 2.**
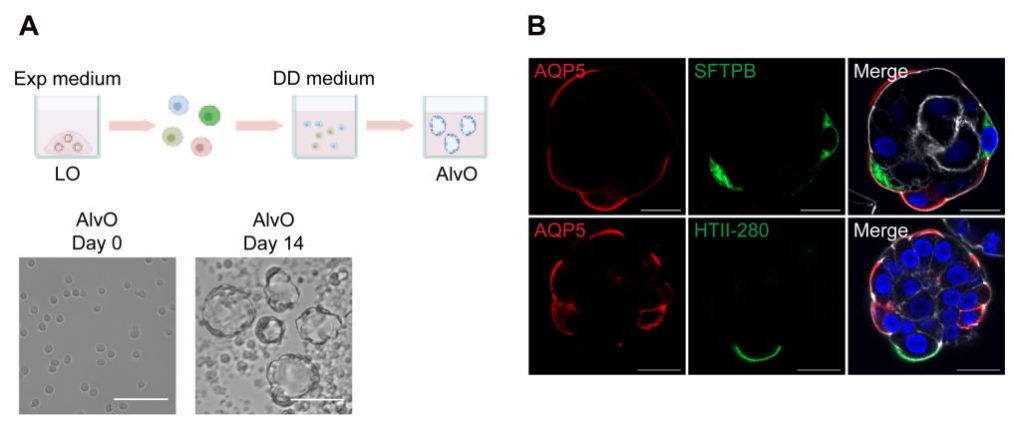
Generation of alveolar organoids. (A). Top, a schematic graph outlines the distal differentiation protocol to generate alveolar organoids (AlvO) in distal differentiation medium (DD medium). Exp medium = expansion medium. Bottom, photomicrographs show single cell suspension on day 0 and mature alveolar organoids on day 14. Scale bar = 100 μm. (B). Alveolar organoids were applied to immunofluorescence staining to label AQP5+ AT1 cells (red) and SFTPB+/HTII-280+ AT2 cells (green). Nuclei and actin filaments were counterstained with DAPI (blue) and Phalloidin-647 (white), respectively. Scale bar = 20 μm.
- **Proximal differentiation of lung organoids to generate optimized 2D airway organoids**
  1. Preparation
    - Prepare proximal differentiation medium (PD medium) (Recipe 4, no PIPES, pH 7.4) and prewarm at 37 °C.
    - Prepare proximal differentiation medium (PD medium) (Recipe 4, with PIPES, pH 6.6) and prewarm at 37 °C.
    - Pre-incubate the Transwell inserts with basal medium overnight in a standard cell culture incubator. For a 24-well insert, add 200 and 500 μL of basal medium to the top and bottom chamber, respectively.
  2. Proximal differentiation in Transwell inserts
    - Culture lung organoids in the expansion medium for 12–14 days after passaging to prepare sufficient cells for differentiation culture (lung organoids from step A3j).
    - Dissociate lung organoids into single cells according to steps B2b–B2g.
    - Resuspend the pellet in 1–4 mL of expansion medium based on the cell number. Count the number of cells with a hemocytometer. Adjust the cell concentration to 1.3 × 10^6^ per milliliter with expansion medium.
    - Remove basal medium from the top and bottom chambers of the pre-incubated Transwell inserts. Seed 100 μL of cell suspension (1.3 × 10^5^ cells) to the top chamber of 24-well inserts. Add 500 μL of expansion medium to the bottom chamber. Incubate the cells in a standard cell culture CO_2_ incubator for two days to let cells grow and attach.
*Note: Approximately 3.12 × 10^6^ cells are required for generating a plate of 2D airway organoids in 24-well Transwell inserts.*
    - Replace the expansion medium with PD medium (pH 7.4) in both the top and bottom chambers. Incubate the cells in a standard cell culture CO_2_ incubator for two days to let cells reach confluence.
*Note: The 2D organoids reach confluence after changing from expansion medium to PD medium (pH 7.4) for 2–3 days.*
    - Replace the PD medium pH 7.4 with PD medium pH 6.6 in the top chamber, while keeping the PD medium pH 7.4 in the bottom chamber (i.e., pH 6.6/7.4 on top/bottom). Incubate the cells in a standard cell culture CO_2_ incubator for 10 days for maturation (Figure 3). Replenish the PD medium pH 6.6 in the top chamber and PD medium pH 7.4 in the bottom chamber every 2–3 days.
*Note: Motile beating cilia are normally discernible in the organoids under a light microscope from day 7 after differentiation in PD medium. After 14 days of proximal differentiation culture, the 2D airway organoids are ready for experimental manipulation.*
    **Figure 3.**
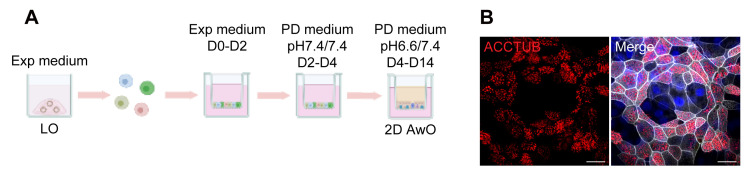
Generation of optimized 2D airway organoids. (A). A schematic graph outlines the proximal differentiation protocol to generate 2D airway organoids (AwO) in proximal differentiation medium (PD medium) at different pH. Exp medium = expansion medium. (B). 2D optimized airway organoids were applied to immunofluorescence staining to label ACCTUB+ ciliated cells (red). Nuclei and actin filaments were counterstained with DAPI (blue) and Phalloidin-647 (white), respectively. Scale bar = 20 μm.
- **Characterization of organoids**
  1. Examine mRNA expression of cell type–specific genes or other cellular genes
    RT-qPCR assay: add 350 μL of cell lysis buffer to harvest the organoids for RNA extraction, reverse transcription, and detection of cellular gene expression.
*Note: Use the autologous undifferentiated lung organoids for comparison/control.*
  2. Assess protein expression of cell type–specific markers and other cellular proteins
    - Immunofluorescence staining and imaging
      i. Fix the organoids with 1 mL of 4% PFA at room temperature for 60 min. Remove the PFA and wash the organoids once with 2% FBS/PBS.
      ii. Permeabilize the organoids with 1 mL of 0.1% Triton X-100 at room temperature for 10 min. Remove the Triton X-100 and wash the organoids once with 2% FBS/PBS.
      iii. Block the organoids with 1 mL of 3% BSA at room temperature for 60 min. Remove the BSA (no need to wash).
      iv. Incubate the organoids with primary antibodies diluted in 2% FBS/PBS at 4 °C overnight. Remove the antibodies and wash them with 2% FBS/PBS five times.
*Note: Refer to the product datasheet for the optimal dilution of the antibody.*
      v. Incubate the organoids with secondary antibodies diluted in 2% FBS/PBS at 4 °C overnight. Remove the antibodies and wash the organoids with 2% FBS/PBS five times.
*Note: Avoid exposure to light after addition of fluorescent materials (i.e., secondary antibody).*
      vi. Counterstain the organoids with DAPI and Phalloidin diluted in 2% FBS/PBS at 4 °C overnight. Remove the dyes and wash the organoids once with 2% FBS/PBS.
      vii. Mount the organoids on a glass slide with glass coverslip using Prolong glass antifade mountant and proceed to confocal imaging.
*Note: The glass slide can be stored at 4 °C in the dark for ~1–2 weeks.*
    - Flow cytometry analysis
      i. Incubate the organoids in 1 mL of 10 mM EDTA at 37 °C for 30–60 min.
      ii. Shear the organoids into single cells by pipetting up and down 20–40 times using a Pasteur pipette.
      iii. Fix the cells with 1 mL of 4% PFA at room temperature for 15–30 min. Remove the PFA and wash the cells once with 2% FBS/PBS.
      iv. Permeabilize the cells with 1 mL of 0.1% Triton X-100 at 4 °C for 5 min. Remove the Triton X-100 and wash the cells once with 2% FBS/PBS.
      v. Incubate the cells with primary antibodies diluted in 2% FBS/PBS at 4 °C for 60 min. Remove the antibodies and wash the cells once with 2% FBS/PBS.
*Note: Refer to the product datasheet for the optimal dilution of the antibody.*
      vi. Incubate the cells with secondary antibodies diluted in 2% FBS/PBS at 4 °C for 60 min. Remove the antibodies and wash the cells once with 2% FBS/PBS.
*Note: Avoid exposure to light after addition of fluorescent materials (i.e., secondary antibody).*
      vii. Resuspend the cells in 2% FBS/PBS at a density of 1 *×* 10^6^ cells per milliliter and transfer the cells to a 5 mL round bottom polystyrene flow tube. Proceed to flow cytometry.
  3. Others
    - Electron microscopy (EM) imaging:
      Fix the organoids with 1 mL of 2.5% glutaraldehyde overnight, followed by EM processing (sectioning and staining) for ultrastructural imaging.
      *Note: Please refer to the detailed protocol provided by the Electron Microscope Unit of HKU (**https://emunit.hku.hk/documents/SamplePreparationTechnique.pdf*).
    - Live cell staining and imaging to assess AT2 cell functionality in alveolar organoids:
      i. Resuspend the alveolar organoids in 0.5 mL of DD medium supplemented with 1 μM of β-BODIPYTM FL C12-HPC. Incubate the organoids at 37 °C overnight in a standard cell culture incubator.
*Note: Avoid exposure to light after the addition of fluorescent materials.*
      ii. Collect the alveolar organoids into a 15 mL centrifuge tube. Wash the organoids with 10 mL of basal medium. Centrifuge at 300 *× g* for 5 min. Discard the supernatant.
      iii. Resuspend the organoids in 0.5 mL of DD medium supplemented with 100 nM of LysoTracker red. Incubate the organoids at room temperature for 30 min.
      iv. Repeat the washing (step D3b.ii).
      v. Mount the live organoids onto a glass slide and proceed to confocal imaging immediately.

## Data analysis

For the related downstream characterization and analysis of organoids such as gene expression, immunofluorescence staining, flow cytometry, electron microscopy, and functional assessment, please refer to our publications (Zhou et al., 2018; Chiu et al., 2022).

## Notes

1. All procedures were conducted in a biosafety cabinet.
2. The protocols were reproducible and have been applied to generate lung organoids and differentiated alveolar/airway organoids derived from multiple donors in our laboratory.
3. The organoids derived from different donors may have a certain degree of variation due to individual genotypic and phenotypic variation (e.g., genes, age, health status).
4. The lung organoids could be cryopreserved for long-term storage and biobanking.
5. The conditioned medium (Rspondin1, Noggin, and WNT3A) can be replaced by commercially available recombinant proteins.
6. The expansion and differentiation media (Recipes 2–4) remain stable for use for approximately 2–4 weeks if properly stored at 4 °C. Prepare fresh medium for optimal performance.

## Recipes

1. **Basal medium**
  **Table:** 

Reagent	Final concentration	Amount
Advanced DMEM/F-12	n/a	500 mL
HEPES (1 M)	10 mM	5 mL
GlutaMAX (200 mM) Penicillin-Streptomycin (10,000 U/mL)	2 mM 100 U/mL	5 mL 5 mL
Total	n/a	515 mL
2. **Expansion medium**
  **Table:** 

Reagent	Final concentration	Amount
Basal medium	n/a	Top up to 100 mL
Rspondin1 Noggin B-27 supplement (50×) N-Acetyl-L-cysteine (500 mM) Nicotinamide (1 M) Y-27632 (50 mM) A 83-01 (500 μM) SB 202190 (10 mM) FGF-7 (5 μg/mL) FGF-10 (100 μg/mL) Primocin (50 mg/mL) Heregulin β-1* (10 μM)	10% 10% 1× 1.25 mM 10 mM 5 μM 500 nM 1 μM 5 ng/mL 20 ng/mL 100 μg/mL 5 nM	10 mL 10 mL 2 mL 250 μL 1 mL 10 μL 100 μL 10 μL 100 μL 20 μL 200 μL 50 μL
Total	n/a	100 mL
  *Only required for initial derivation of organoids (first passage)
3. **Distal differentiation medium (DD medium)**
  **Table:** 

Reagent	Final concentration	Amount
Basal medium	n/a	Top up to 100 mL
Dexamethasone (10 μM) 8-Bromo-cAMP (100 mM) IBMX (50 mM) B-27 supplement (50×) Primocin (50 mg/mL) WNT3A* CHIR99021* (10 mM)	50 nM 100 μM 100 μM 1× 100 μg/mL 50% 3 μM	500 μL 100 μL 200 μL 2 mL 200 μL 50 mL 30 μL
Total	n/a	100 mL
  *Add either 50% WNT3A or 3 μM CHIR99021
4. **Proximal differentiation medium (PD medium)**
  **Table:** 

Reagent	Final concentration	Amount
PneumaCult-ALI Basal Medium	n/a	450 mL
PneumaCult-ALI 10× Supplement	1×	50 mL
PneumaCult-ALI Maintenance Supplement (100×) Heparin (2 mg/mL) Hydrocortisone (96 μg/mL) DAPT (10 mM) Primocin (50 mg/mL) PIPES*# (1 M)	1× 4 μg/mL 480 ng/mL 10 μM 100 μg/mL 2 mM	5 mL 1 mL 2.5 mL 500 μL 1 mL 1 mL
Total	n/a	510 mL
  *Prepare two PD media, one with PIPES and one without
  #For the PD medium with PIPES, adjust the pH to 6.6 with HCl and NaOH after addition of PIPES
5. **Rspondin1 conditioned medium**
  - Prepare selection medium (500 mL of DMEM + 60 mL of FBS + 5 mL of penicillin-streptomycin + 1.5 mL of zeocin selection reagent).
  - Prepare growing medium (500 mL of DMEM + 60 mL of FBS + 5 mL of penicillin-streptomycin).
  - Culture the Rspondin1 expressing 293T cells in a T175 flask in selection medium in a standard cell culture CO_2_ incubator and wait until they reach confluence.
  - Split the cells into six T175 flasks in growing medium and wait until they reach confluence.
  - Remove the growing medium and culture the cells in 50 mL basal medium (Recipe 1) for seven days.
  - Collect the medium (i.e., the Rspondin1 conditioned medium). Centrifuge at 300 *×* g for 5 min to pellet the cells/cell debris and filter the medium through a 0.22 μm bottle top vacuum filter.
  - Aliquot the medium into small volumes (~50 mL) and store them at -80 °C.
6. **Noggin conditioned medium**
  - Prepare selection medium (500 mL of DMEM + 60 mL of FBS + 5 mL penicillin-streptomycin + 5 mL of G418 sulfate).
  - Prepare growing medium (500 mL of DMEM + 60 mL of FBS + 5 mL of penicillin-streptomycin).
  - Culture the Noggin expressing HEK293 cells in a T175 flask in selection medium in a standard cell culture CO_2_ incubator and wait until they reach confluence.
  - Split the cells into six T175 flasks in growing medium and wait until they reach confluence.
  - Remove the growing medium and culture the cells in 50 mL of basal medium (Recipe 1) for seven days.
  - Collect the medium (i.e., the Noggin conditioned medium). Centrifuge at 300 *×* g for 5 min to pellet the cells/cell debris and filter the medium through a 0.22 μm bottle top vacuum filter.
  - Aliquot the medium into small volumes (~50 mL) and store them at -80 °C.
7. **WNT3A conditioned medium**
  - Prepare selection medium (500 mL of DMEM + 60 mL of FBS + 5 mL penicillin-streptomycin + 625 μL of zeocin).
  - Prepare growing medium (500 mL of DMEM + 60 mL of FBS + 5 mL of penicillin-streptomycin).
  - Culture the Rspondin1 expressing 293T cells in a T175 flask in selection medium in a standard cell culture CO_2_ incubator and wait until they reach confluence.
  - Split the cells into six T175 flasks in growing medium and wait until they reach confluence.
  - Split the cells into thirty 100 mm cell culture dishes in growing medium (20 mL per dish) and culture the cells for seven days.
  - Collect the medium (i.e., the WNT3A conditioned medium). Centrifuge at 300 *×* g for 5 min to pellet the cells/cell debris and filter the medium through a 0.22 μm bottle top vacuum filter.
  - Aliquot the medium into small volumes (~50 mL) and store them at -80 °C.
